# 

**Published:** 1890-05-17

**Authors:** 


### NEW OFFICES IN THE STRAND.

The Hospital *is now published at* 140, Strand, W.C. *It has been found desirable to establish* The Hospital *in this central and convenient locality, owing to the rapid extension of the business of this paper of late.*

### NOTICE TO CORRESPONDENTS.

All MS., Letters, Books for Review, and other matters intended for the Editor should be addressed The Editor, The Lodge, Porchester Square, London, W.

The Editor cannot undertake to return rejected M.S., even when accompanied by stamped directed envelope.

### Notice to Advertisers and Subscribers.

All Advertisements, Orders for Copies of The Hospital, and Business Communications should be addressed to The Publisher, *not to the Editor*, The Hospital, 140, Strand, W.C.

### Bound Volumes of "The Hospital."

Vol. VI., containing full page portraits of T.R.H. the Prince and Princess of Wales, and Miss Florence Nightingale, is obtainable at 4s., post free.

Orders will be received for Vol. VII., 4s. post free. Cases for binding may be had of the Publisher, at 1s. 6d. each.

### To Residents, Secretaries, and Matrons.

The Editor will be glad to have the Regulations and each Report as issued by Asylums, Hospitals, Convalescent and Nursing Institutions, as well as those of other Charities, and an early copy in duplicate of all Appeals and Festival Notices, etc., addressed to *The Editor, The Lodge, Porchester Square, London, W.* Any superintendent, secretary, matron, or other chief official who regularly sends such information may be registered, on application to the Publisher, to receive free of cost a cover for binding The Hospital in half-yearly volumes.

### Scraps and Gleanings.

There is to be an ambulance company to every Volunteer brigade in future.

Mr. Junius S. Morgan has left 20,000 dollars to Hartford Hospital, Connecticut.

Professor Sullivan, the distinguished chemist, died on Monday, at Cork Queen's College.

We regret to hear that Lady Verney, sister of Miss Florence Nightingale, died last Sunday.

Messrs. Cooper gave a private view a day or two ago of the artistic application of electric light.

An inmate of St. Anne Asylum, Paris, lately committed suicide by plunging a pin into her left breast.

A Lunatic at Bethlem Hospital lately wrested a razor from the hands of an attendant, and cut his own throat.

During the nine months that Marlow Cottage Hospital has been opened, 25 patients have been admitted.

Sheffield Infirmary has lost a valued friend by the death of Mr. Brooksbank, who had been on its board for 37 years.

Surgeon Parke has been specially requested, and has consented, to give evidence before the Royal Commission upon Vaccination.

The Convalescent Home at Saltburn-by-Sea continues its good work, and nearly 500 patients were admitted last year. For terms apply to the matron.

Mr. Hampton Hale presided at the annual meeting of the British Home for Incurables. At present there are 55 in-patients and 31 out-patients.

Lord Hartington will preside at the annual meeting of the Children's Country Holidays Fund, at Devonshire House, on Monday afternoon, May 19th.

John Murphy, the old soldier affected with leprosy, whose case caused a good deal of alarm in Dublin a year ago, died recently in the Hardwicke Hospital.

A private subscription dance will be held at Kensington Town Hall on June 3rd, under the patronage of the Duchess of Teck, in aid of the Extension Fund of the West London Hospital.

The Croonian lectures will be delivered at the Royal College of Physicians by Dr. Ferrier, on June 3rd, 10th, 17th, 19th, 24th, and 26th. The subject will be "Cerebral Localization."

It is stated on medical authority that the influenza epidemic has reappeared in a rather acute form at Warsaw, and that in the environs of that city the disease has even attacked horses.

The Duchess of Albany presided at a drawing-room meeting on Tuesday, when Sir Douglas Galton spoke on the new scheme for the Education of Women in Personal and Domestic Hygiene.

The Ecclesiastical Commissioners have just sent £500 towards clearing off the debt of the Sunderland Infirmary, and Lord Londonderry has also sent a donation of £100 towards the same object.

The officers of the Army Medical Staff, past and present, entertained Surgeon Parke at a dinner on his return from the Emin Pacha Relief Expedition, in the Whitehall Rooms, Hôtel Métropole, last Monday.

Lady Wimborne is about to add to the long list of charitable organizations in Dorsetshire, of which she is the patroness and founder, by arranging a new cottage hospital at Poole for the benefit of the poor of the town.

The Paris Municipal Council has voted the construction of an asylum for pregnant women of the poorer classes who by reason of their pregnancy are rendered for the time being incapable of following their avocations.

The Theatrical Mission annual festival will take place on Saturday afternoon, May 17, at 3.30, when the Duchess of Teck has consented to be present, and to receive purses in aid of the completion of the purchase of Macready House.

The *Daily News* tells of some very big fees given to the Russian surgeon, whose lengthy name we spare our readers. Still, a fee of over £1,000 for an operation in a case of abscess of the hip would console one for a name all consonants.

The Queen has forwarded another donation of £50 in aid of the funds of the Railway Benevolent Institution, through the Marquis of Tweeddale, who presided at the annual dinner of the society on the 2nd inst. This is her Majesty's sixth donation.

The Queen has forwarded a donation of £50 in aid of the funds of the Railway Benevolent Institution, through the Marquis of Tweeddale, who presided at the annual dinner of the society on the 2nd inst. This is her Majesty's sixth donation to the institution.

The Lord Mayor has received an intimation that the late Mr. Thomas Crouch of New Union-street, Cripplegate, has left to the Hospital Sunday Fund the sum of £1,003, without deducting 10 per cent. legacy duty, which will probably be refunded.

The Austrian Government, having asked for medical reports on the disease called "Nona," has become satisfied that this complaint is only a form of neurosis which affects persons of nervous and debilitated constitutions, but chiefly those suffering from anæmia.

The will of the late Miss Jane Wilson, of Belgrave-place, has been proved, the personal estate amounting to over £56,000. She has left £14,000 to the S.P.G. for general purposes, another large sum for the work of the Society in India, and a considerable legacy to St. George's Hospital.

In the quarterly report of the executive committee of the Middlesex County Council the number of rabid dogs is stated to have amounted to seven, as against two in the corresponding quarter of last year. During the six months ending March, eighteen dogs were destroyed as suffering from rabies.

Sir T. Fowell Buxton has been informed that Mr. J. Passmore Edwards has decided to give £20,000 to the extension fund now being raised for the erection and endowment of the Bethnal green Free Library, provided that the other £20,000 required are raised by public subscription.

